# Altered ERK1/2 Signaling in the Brain of Learned Helpless Rats: Relevance in Vulnerability to Developing Stress-Induced Depression

**DOI:** 10.1155/2016/7383724

**Published:** 2015-12-29

**Authors:** Yogesh Dwivedi, Hui Zhang

**Affiliations:** Department of Psychiatry and Behavioral Neurobiology, University of Alabama at Birmingham, Birmingham, AL 35294, USA

## Abstract

Extracellular signal-regulated kinase 1/2- (ERK1/2-) mediated cellular signaling plays a major role in synaptic and structural plasticity. Although ERK1/2 signaling has been shown to be involved in stress and depression, whether vulnerability to develop depression is associated with abnormalities in ERK1/2 signaling is not clearly known. The present study examined ERK1/2 signaling in frontal cortex and hippocampus of rats that showed vulnerability (learned helplessness, (LH)) or resiliency (non-learned helplessness, (non-LH)) to developing stress-induced depression. In frontal cortex and hippocampus of LH rats, we found that mRNA and protein expressions of ERK1 and ERK2 were significantly reduced, which was associated with their reduced activation and phosphorylation in cytosolic and nuclear fractions, where ERK1 and ERK2 target their substrates. In addition, ERK1/2-mediated catalytic activities and phosphorylation of downstream substrates RSK1 (cytosolic and nuclear) and MSK1 (nuclear) were also lower in the frontal cortex and hippocampus of LH rats without any change in their mRNA or protein expression. None of these changes were evident in non-LH rats. Our study indicates that ERK1/2 signaling is differentially regulated in LH and non-LH rats and suggests that abnormalities in ERK1/2 signaling may be crucial in the vulnerability to developing depression.

## 1. Introduction

Depression is a debilitating psychiatric illness with a lifetime prevalence rate of about 5–20% [1–3]. A large number of depressed patients do not respond to antidepressants and a majority of them show resistance to treatment [4, 5]. This could partially be due to a lack of understanding of the molecular mechanisms associated with the etiology and pathogenesis of depression.

In recent years, the hypothesis that depression is associated with altered gene-environment interaction and impaired synaptic and structural plasticity has gained significant attention [6–9]. Extracellular signal-regulated kinases 1/2 (ERK1/2) signaling, which belongs to a large family of mitogen-activated protein kinase signaling cascades, has consistently been shown to have a major impact on both synaptic plasticity and structural plasticity. This is evident from studies showing their role in long-term potentiation, long-term depression, and the regulation of neuronal survival via neurotrophic/growth factors [10–12]. In this signaling pathway, ERK1 and ERK2 are the two major components. Both ERK1 and ERK2 are activated by upstream mitogen-activated protein kinase kinases MEK1 and MEK2 via phosphorylation at threonine and tyrosine residues within their activation loop [13]. This phosphorylation facilitates transduction of extracellular signals from cell surface receptors to the nucleus because phosphorylated ERK1 and ERK2 are translocated from cytosol to nucleus where they further phosphorylate target proteins and inhibit or activate transcription of a large number of genes [14]. Activated ERK1 and ERK2 can also affect the functions of various proteins within the cytosol. Interestingly, because of a high homology in their amino acid sequences, ERK1 and ERK2 share several common substrates [15, 16] that regulate neuronal excitability, histone modifications, synaptogenesis, and cell cycle [17–23], which thus participate in behavioral and cognitive processes [24–26]. ERK1/2 signaling is terminated via dephosphorylation by “dual function” MAP kinase phosphatases [27, 28].

Because ERK1 and ERK2 regulate synaptic plasticity and structural plasticity, in recent years, several studies have focused their possible role in stress-related disorders such as depression. We were the first to demonstrate that ERK1/2 signaling was hypoactive in the frontal cortical (Brodmann areas 8, 9, and 10) and hippocampal brain areas of depressed patients [29–31]. Recently, an integrated transcriptome analysis derived from rat and human prefrontal cortex has identified ERK1/2 as one of the leading signaling kinases to be highly associated with depression [32]. At the behavioral level, ERK1 ablation in mice causes hyperactivity and resistance to behavioral despair [33, 34] and treatment of rats with MEK inhibitor induces mood disorder-related behavioral deficits [35]. On the other hand, peripheral injection of MEK inhibitor eliminates the response to antidepressants in behavioral despair [35]. Since adaptive/maladaptive response to stress is crucial in inducing depression, it is interesting to examine whether vulnerability or resiliency to developing depression is associated with differential regulation of ERK1/2 signaling.

To do so, in the present study, we used an animal model of depression that can distinguish vulnerability or susceptibility to developing stress-induced depression. This model is based on proactive interference with the acquisition of escape or avoidance response when animals are subjected to unpredicted and uncontrollable stress [36]. In this model, which is termed as learned helplessness (LH) model of depression, rodents show emotional, cognitive, and motivational deficits. On the other hand, the non-learned helpless animals (non-LH, resilient), although given the same uncontrollable and unpredictable stress, fail to show such responses. This provides an opportunity to distinguish the neurobiological factors associated with resiliency versus vulnerability to developing depression. In our earlier studies, we had modified the stress paradigm in such a way that it significantly prolonged the duration of depressive behavior from 24 hours to 14 days [37–39]. This is quite advantageous in examining the factors associated with chronic depression. Using this animal model, we explored whether ERK1/2 signaling plays a role in developing depressive behavior. For this, in the frontal cortex and hippocampus of LH, non-LH, and tested control (TC) rats, we determined the activation and expression of ERK1 and ERK2 at both transcriptional and translational levels. The activation of ERK1 and ERK2 was determined in cytosolic and nuclear fractions by examining expression levels of phosphorylated ERK1 and ERK2 and their mediated phosphorylation of substrate Elk1. In addition, we examined functional significance of altered ERK1 and ERK2 by determining activation and expression of their downstream common substrates RSK (90 kDa S6 kinase) and MSK (mitogen and stress-activated kinase).

## 2. Materials and Methods

Male Sprague-Dawley rats (Holtzman strain) weighing between 325 and 370 g were obtained from Harlan Sprague-Dawley Laboratories, USA. Rats were placed at 21 ± 1°C temperature and 55 ± 5% humidity. Initially, during acclimatization, rats were placed randomly (3/cage); however, after initial behavioral testing, they were grouped according to their behavioral phenotype. The light and dark cycle was 12 hours. Rats were given ad libitum food and water. The rats were acclimatized for two weeks prior to the start of the shock paradigm. All the behavioral experiments were performed between 8 and 10 am. The protocol to induce learned helpless behavior was approved by the Institutional Animal Care and Use Committee of the University of Alabama at Birmingham. All the experiments were done in 6 LH, 6 non-LH, and 6 TC rats.

### 2.1. Induction of Learned Helpless Behavior

The protocol for the induction of learned helpless behavior has been described in great detail in our earlier publications [37–39]. Briefly, rats were subjected to 100 random inescapable tail shocks (IS) at the intensity of 1.0 mAmp for 5 seconds. The average interval between two shocks was 60 seconds. Escape latency was determined 24 hours later. These rats were given additional IS on day 7 and tested for escape latency on day 8 and again on day 14. Another group of rats were tested for escape latency without giving any shock. These rats were termed as tested control (TC). The escape latency was tested using two different trials: FR1 and FR2. In FR1 (5 trials), rats were given foot shock at the intensity of 0.6 mAmp at variable time intervals. The rats had to escape the foot shock by moving from one chamber to another. In FR2 (25 trials), the rats had to cross from one chamber to the other and had to come back to the original chamber to terminate the shock. The shocks were terminated automatically after 30 sec. Escape latencies were automatically recorded through computer generated programs (Med Associates, USA). All the rats were sacrificed 24 hours after the last escape latency test. Rats were decapitated and blood was collected for plasma corticosterone levels (Abcam, USA). Various brain areas were dissected immediately and kept at −80°C for analyses. Based on escape latency in FR2 trial, rats were divided into two groups: learned helpless (LH, showing escape latency ≥20 seconds) and non-learned helpless (non-LH, showing escape latency <20 seconds). Generally, the rats who showed LH behavior in the FR2 trial (day 2) remained LH throughout the experimental duration (day 14). We found almost equal distribution of rats among LH and non-LH groups.

### 2.2. Isolation of Cytosolic and Membrane Fractions

These fractions were isolated at 4°C as previously described [37]. Briefly, frontal cortex and hippocampus were homogenized in 10 mM HEPES buffer (pH 7.4), containing various protease and phosphatase inhibitors (NaF [50 mM], phenylmethylsulfonyl fluoride [1 mM], ethylene glycol tetraacetic acid, EGTA [1 mM], sodium orthovanadate [2 mM], ethylenediaminetetraacetic acid, EDTA [1 mM], sodium pyrophosphate [10 mM], para-nitrophenylphosphate [4 Mm], leupeptin [10 *μ*g/mL], pepstatin A [10 *μ*g/mL], aprotinin [4 *μ*g/mL], and NP-40 [0.5%]). The homogenate was centrifuged at 12,000 ×g for 1 hour. The supernatant was again centrifuged at 100,000 ×g for 1 hour. The pellet was the membrane fraction, whereas the supernatant was used as cytosolic fraction. The pellet containing the membrane fraction was suspended in Tris-HCl buffer (50 mM, pH 7.5) containing various protease and phosphatase inhibitors as described above. The protein contents were measured by Lowry et al. [40].

### 2.3. Isolation of Nuclear Fraction

Frontal cortex and hippocampus were homogenized in a 10 mM HEPES buffer (pH 7.4) containing various protease and phosphatase inhibitors as detailed above and spun at 100,000 ×g for 30 min. The pellet was again homogenized in 20 mM HEPES buffer (pH 7.4) containing glycerol (50%), NaCl (84 mM), MgCl_2_ (1.5 mM), EDTA (0.4 mM), and various protease inhibitors, and it was kept at 4°C for 15 min while shaking. The suspension was centrifuged at 20,000 ×g for 15 min. As described earlier [37], the purity of these fractions was confirmed using antibodies against histone H2B (nuclear) and PKA RII subunit (cytosolic) (data not shown).

### 2.4. ERK1 and ERK2 Assay

The determination of catalytic activities of ERK1 and ERK2 was performed by the procedure described earlier [29]. The active ERK1 and ERK2 were immunoprecipitated using p-ERK1/2 antibody (Santa Cruz Biotechnology, USA). The immunoprecipitation technique was followed as discussed in our earlier publication [41]. Protein A Sepharose beads were used to pull down active ERK1 and ERK2 followed by centrifugation (2,500 rpm for 30 min) and the suspension was washed twice with 20 mM Tris lysis buffer (pH 7.5) containing various phosphatase and protease inhibitors and twice with 25 mM Tris (pH 7.5) kinase buffer containing *β*-glycerophosphate (5 mM), 2 mM dithiothreitol (2 mM), sodium orthovanadate (0.1 mM), and MgCl_2_ (10 mM). The kinase reaction was initiated with suspending the pellet in kinase buffer containing ATP (200 *μ*M) and Elk1 fusion protein (2 *μ*g; GST fused to Elk1 codons 307–428). The reaction was carried out for 30 minutes at 30°C and terminated by adding Laemmli buffer. Samples were run for gel electrophoresis followed by transfer to nitrocellulose membrane and incubation with primary (p-Elk1; Santa Cruz Biotechnology, USA) and HRP-conjugated anti-rabbit secondary antibody. Each membrane was stripped and reprobed with *β*-actin primary antibody (Sigma Chemical Co., USA) and anti-mouse secondary antibody. The optical density of each band was calculated using software provided by Loats Image Analysis System, USA. A ratio of the optical densities of Elk1 and corresponding *β*-actin band was determined.

### 2.5. mRNA Expression of ERK1, ERK2, RSK1, and MSK1 by Quantitative Real-Time PCR

RNA from each sample was isolated using TRIzol (Life Technologies, USA), and purity ratios (260/280 nm and 260/230 nm) were assessed by NanoDrop (Thermo Scientific). TaqMan primers and probe sets (Life Technologies, USA) were used for qRT-PCR. The methods for qRT-PCR were followed as described in the manufacturer's protocol and discussed in our earlier publication [39]. *β*-actin was used as endogenous control (normalizer). Fold changes were calculated using 2^−ΔΔCt^ method [39].

### 2.6. Immunolabeling of ERK1 and ERK2 and total and ERK1/2-Mediated Phosphorylation of RSK1 and MSK1

Expression levels of total ERK1 and ERK2 were determined in total tissue lysates, whereas total and phosphorylated RSK1 were determined in both the cytosolic and nuclear fractions by Western blot [29, 30]. The levels of total and p-MSK1 were determined in the nuclear fraction. For ERK1/2-mediated phosphorylation of RSK1 and MSK1, tissue samples were immunoprecipitated with p-ERK1/2 antibody as discussed above. Samples containing 25 *μ*g of protein were subjected to gel electrophoresis followed by transfer to nitrocellulose membrane. Following were the primary antibodies used: ERK1, ERK2 (Santa Cruz Biotechnology, USA), RSK1 (Abcam, USA), MSK1 (Santa Cruz Biotechnology, USA), p-ERK1/ERK2 (Santa Cruz Biotechnology, USA), p-RSK1 (phospho-Ser380; Abcam, USA), and p-MSK1 (phospho-Ser360; Abcam, USA). The dilution for each antibody was as follows: ERK1 (1 : 1000), ERK2 (1 : 1000), RSK1 (1 : 1500), MSK1 (1 : 1000), p-ERK1/ERK2 (1 : 1000), p-RSK1 (1 : 1500), or p-MSK1 (1 : 1000). The nitrocellulose membranes were stripped using a buffer (Chemicon International, USA) and exposed with *β*-actin antibody. The bands on the autoradiograms were determined and ratio of the optical density of the protein of interest to the corresponding *β*-actin band was calculated. The results are given as percent of the control.

### 2.7. Immunoprecipitation and Assay of RSK1 and MSK1 Catalytic Activities

Tissues lysates (containing 100 *μ*g protein) were immunoprecipitated using antibodies for MSK1 or RSK1 as described above. Their catalytic activities were assayed essentially by the procedure described by Sapkota et al. [42]. The immunoprecipitates derived from protein A Sepharose were washed with 50 mM Tris-HCl buffer (pH 7.5) containing EGTA (0.1 mM), 2-mercaptoethanol (0.1% v/v), PKA inhibitor (2.5 *μ*M; TTYADFIASGRTGRRNAIHD), and magnesium acetate (10 mM). The reaction was initiated by adding [*γ*-^32^P]ATP (~1000 cpm/pmol) and Crosstide (GRPRTSSFAEG, 30 *μ*M; Enzo Life Sciences, USA). The assay was terminated after 15 min. One milliunit of activity denotes the amount of enzyme that catalyzes the phosphorylation of 1 pmol Crosstide per minute. The results are provided as percent of control.

### 2.8. Statistical Analysis

Statistical Package for the Social Sciences (SPSS) was used for all the data analysis. The data are represented as mean ± standard deviation (SD). TC, LH, and non-LH groups were compared using one-way ANOVA. Post hoc comparisons were calculated by Tukey's method of multiple comparisons. All the 3 groups were compared among each other: LH group was compared with TC and non-LH groups; non-LH group was compared with LH and TC groups; TC group was compared with LH and non-LH groups. Significance level was set at *P* < 0.05. Correlation analyses were performed using Pearson product moment. Statistical significance levels (overall and individual) are provided in each figure legend.

## 3. Results

### 3.1. Escape Latencies

**Figure 1:**
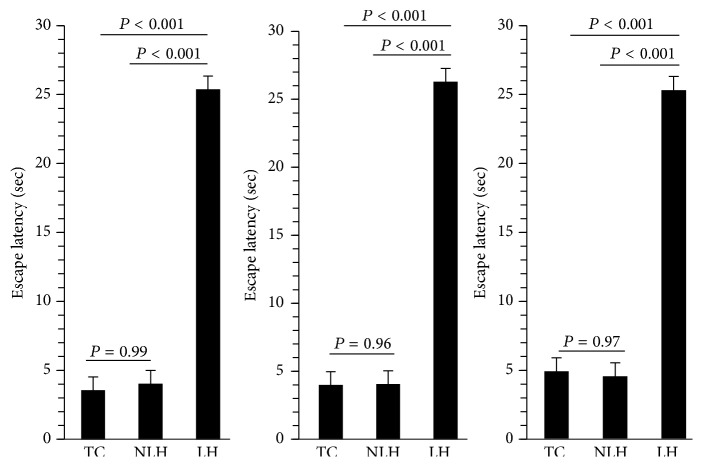
Escape latencies in LH, non-LH, and TC rats measured on days 2, 8, and 14. Data are the mean ± SD from 6 rats in each group. Overall group differences were as follows: day 2: *F* = 2,15, df = 179, and *P* < 0.001; day 8: *F* = 2,15, df = 134, and *P* < 0.001; and day 14: *F* = 2,15, df = 132, and *P* < 0.001. Individual group analysis revealed that, at all these time intervals, LH group was significantly different from NLH or TC groups (*P* < 0.001). There was no significant difference between NLH and TC groups.

As mentioned above, we determined escape latencies on days 2, 8, and 14. The escape latencies in LH rats were significantly greater than non-LH and TC rats at all these 3 time points. There were no significant differences between NLH and TC groups (Figure 1).

### 3.2. Serum Corticosterone Level

**Figure 2:**
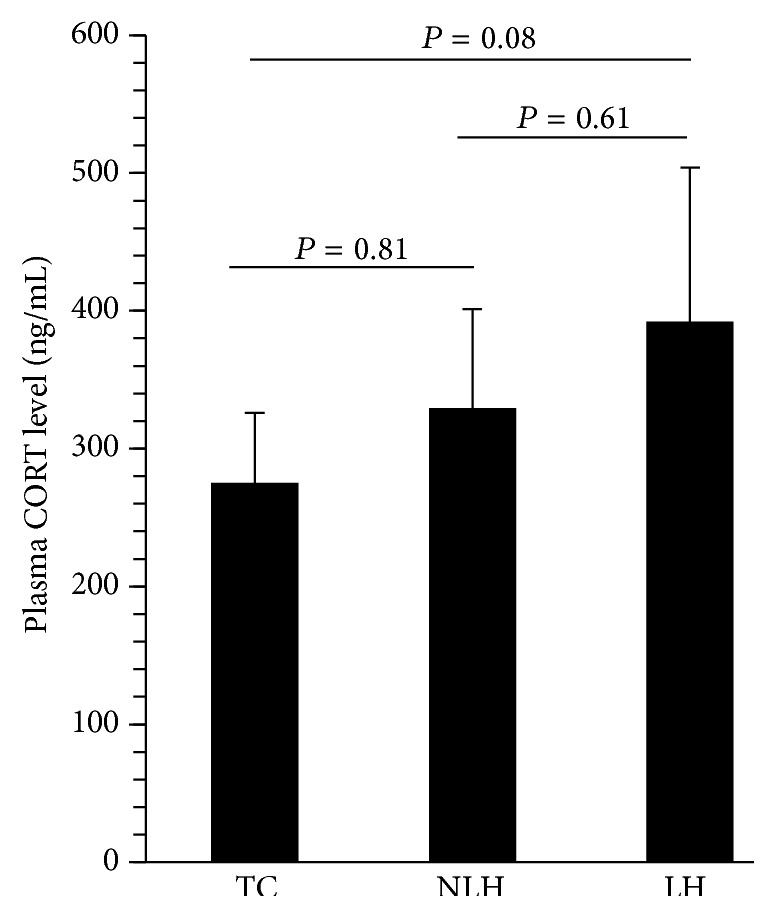
Plasma corticosterone (CORT) levels in TC, non-learned helpless (non-LH), and learned helpless (LH) rats. Data are the mean ± SD from 6 animals per group. The overall group differences were as follows: df = 2,15, *F* = 3.07, and *P* = 0.29.

As indicated in Figure 2, the plasma levels of CORT were not significantly different between non-LH, LH, and TC rats.

### 3.3. Immunolabeling of ERK1 and ERK2

**Figure 3:**
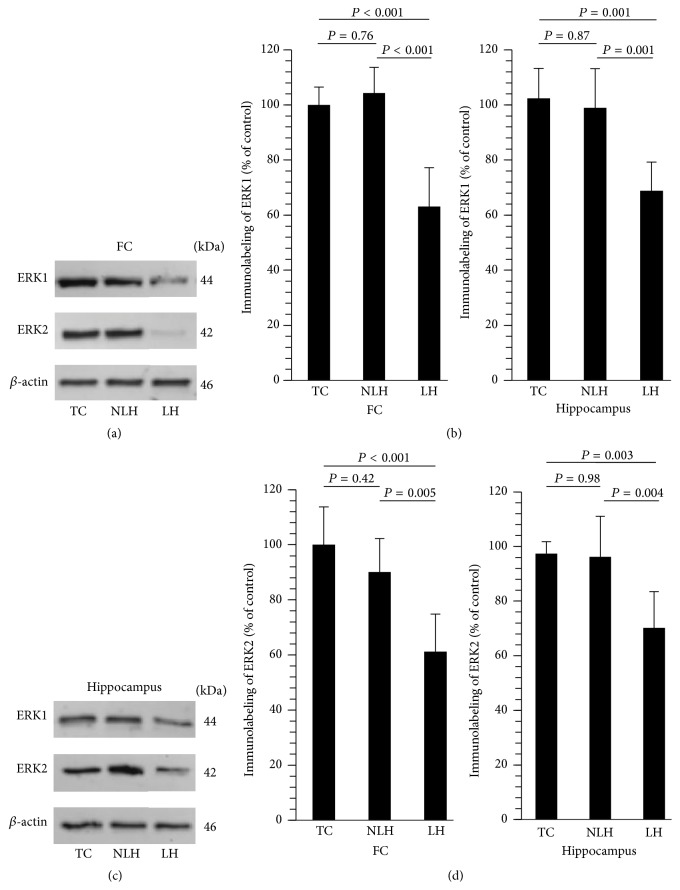
Western blots showing immunolabeling of ERK1 and ERK2 in frontal cortex (FC) (a) and hippocampus (b) of tested control (TC), non-NLH (NLH), and learned helpless (LH) rats. *β*-actin was used as endogenous control and a ratio of optical density of ERK1 and ERK2 to the optical density of the corresponding *β*-actin was calculated. (b) Mean ± SD of protein expression levels of ERK1 and ERK2 in FC (c) and hippocampus (d) of TC, non-LH, and LH rats (*n* = 6/group). Overall group differences in the 3 groups are as follows. ERK1: FC, df = 2,15, *F* = 28.10, and *P* < 0.001; hippocampus, df = 2,15, *F* = 14.35, and *P* < 0.001. ERK2: FC, df = 2,15, *F* = 14.09, and *P* < 0.001; hippocampus, df = 2,15, *F* = 10.16, and *P* < 0.002.

Representative Western blots for ERK1 and ERK2 in frontal cortex and hippocampus of various groups of rats are given in Figures 3(a) and 3(c), respectively. It was found that the expressions of ERK1 and ERK2 were significantly decreased in frontal cortex (Figure 3(b)) and hippocampus of LH rats (Figure 3(d)) when compared with TC and non-LH rats. On the other hand, the levels of ERK1 and ERK2 were similar in TC and non-LH rats in both these brain areas.

### 3.4. mRNA Expression of ERK1 and ERK2

**Figure 4:**
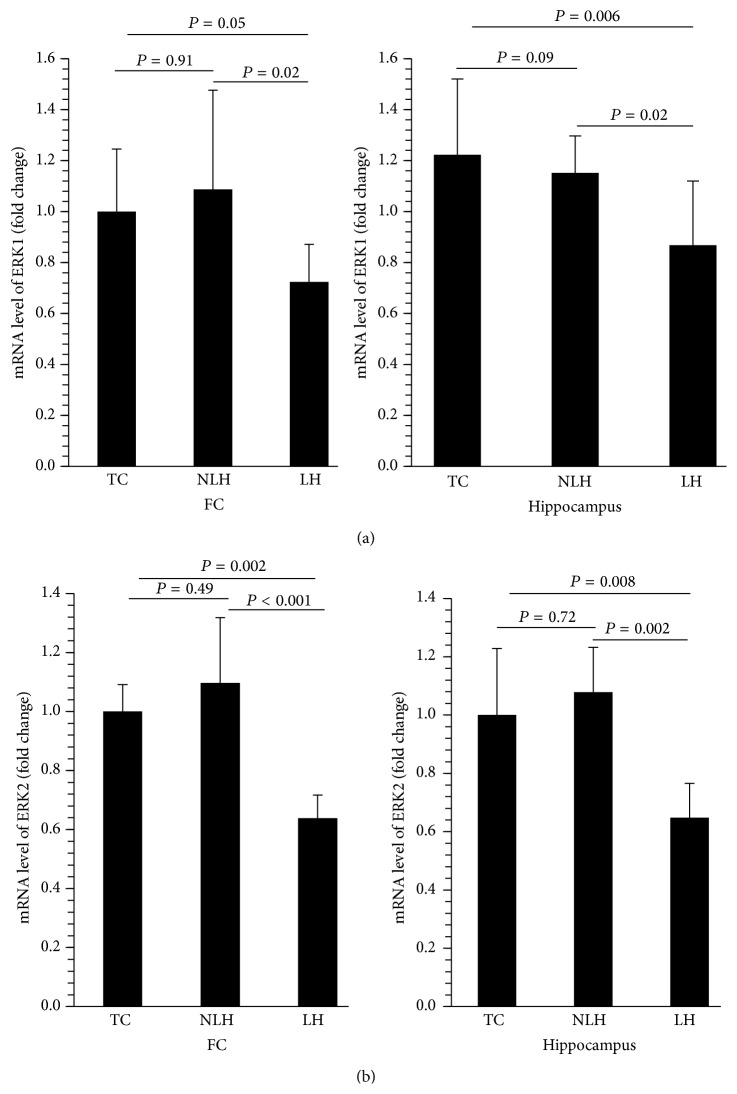
mRNA levels of ERK1 and ERK2 in frontal cortex (FC) (a) and hippocampus (b) of TC, non-LH, and LH rats. Data are the mean ± SD from 6 animals in each group. Overall group differences in the 3 groups are as follows. ERK1: FC, df = 2,15, *F* = 5.17, and *P* = 0.02; hippocampus, df = 2,15, *F* = 7.92, and *P* = 0.004. ERK2: FC, df = 2,15, *F* = 16.53, and *P* < 0.001; hippocampus, df = 2,15, *F* = 10.51, and *P* = 0.001.

The gene expression of these two kinases was determined in the same samples in which their protein levels were examined. As with protein levels, mRNA expressions of ERK1 and ERK2 were significantly decreased in both frontal cortex (Figure 4(a)) and hippocampus (Figure 4(b)) of LH rats, whereas no significant differences were found between non-LH and TC rats.

### 3.5. Catalytic Activities of ERK1 and ERK2

In previous studies, we have characterized ERK1 and ERK2 catalytic activities in cytosol and membrane fractions obtained from human postmortem samples and found that catalytic activities of these kinases reside in the cytosol fraction; in the membrane fraction, their activities were either not detectable or marginally detectable. Similar reports have been shown in previous studies by other investigators [43, 44]. To ensure that similar phenomenon occurs in rat brain, we characterized their activities in the cytosolic and membrane fractions and found similar results (data not shown). Therefore, in subsequent experiments, we determined ERK1 and ERK2 activities in the cytosol fraction. Because there is a translocation of ERK1 and ERK2 from cytosol to nucleus upon phosphorylation, we examined the catalytic activities of ERK1 and ERK2 also in the nuclear fraction.

**Figure 5:**
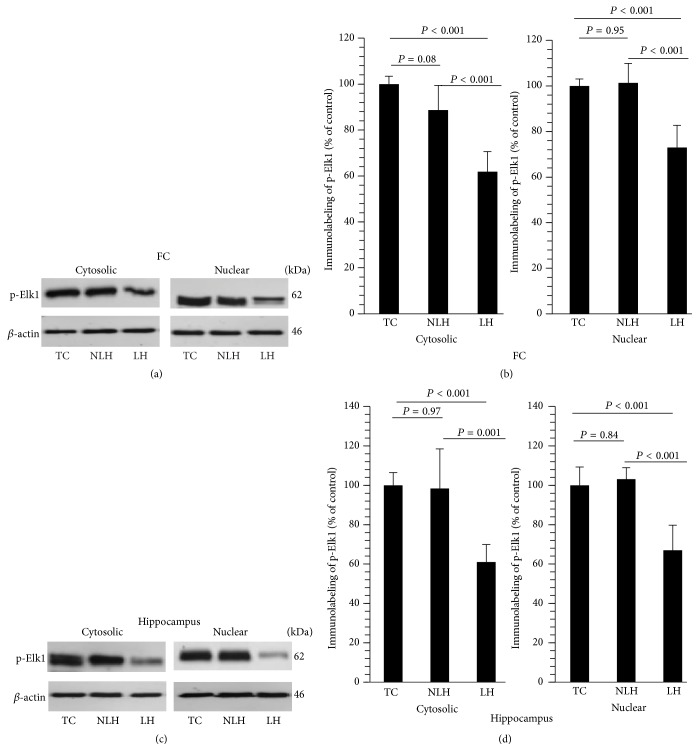
Catalytic activities of ERK1/2 (represented as ERK1/2-mediated phosphorylation of Elk1) in cytosolic and nuclear fractions of FC (a) and hippocampus (c) obtained from TC (tested controls), non-learned helpless (non-LH), and learned helpless (LH) rats. *β*-actin was used as normalizer. Mean ± SD of Elk1 phosphorylation in cytosolic and nuclear fractions of FC (b) and hippocampus (d) of TC, non-LH, and LH rats (*n* = 6/group). Overall group differences among TC, non-LH, and LH rats are as follows. FC: cytosolic, df = 2,15, *F* = 33.71, and *P* < 0.001; nuclear, df = 2,15, *F* = 25.93, and *P* < 0.001. Hippocampus: cytosolic, df = 2,15, *F* = 16.63, and *P* < 0.001; nuclear, df = 2,15, *F* = 25.57, and *P* < 0.001.

Figures 5(a) and 5(c) show representative Western blots depicting ERK1/2 activity (p-Elk1 as a measure of activated ERK1/2) in cytosolic and nuclear fractions of frontal cortex and hippocampus obtained from various groups of rats (TC, non-LH, and LH). The kinase activities of ERK1 and ERK2 were significantly down in cytosolic and nuclear fractions of LH rats both in frontal cortex (Figure 5(b)) and hippocampus (Figure 5(d)). ERK1/2 activity was not altered in non-LH rats compared with TC rats in either cytosol fraction or nuclear fraction.

### 3.6. Phosphorylation of ERK1 and ERK2

**Figure 6:**
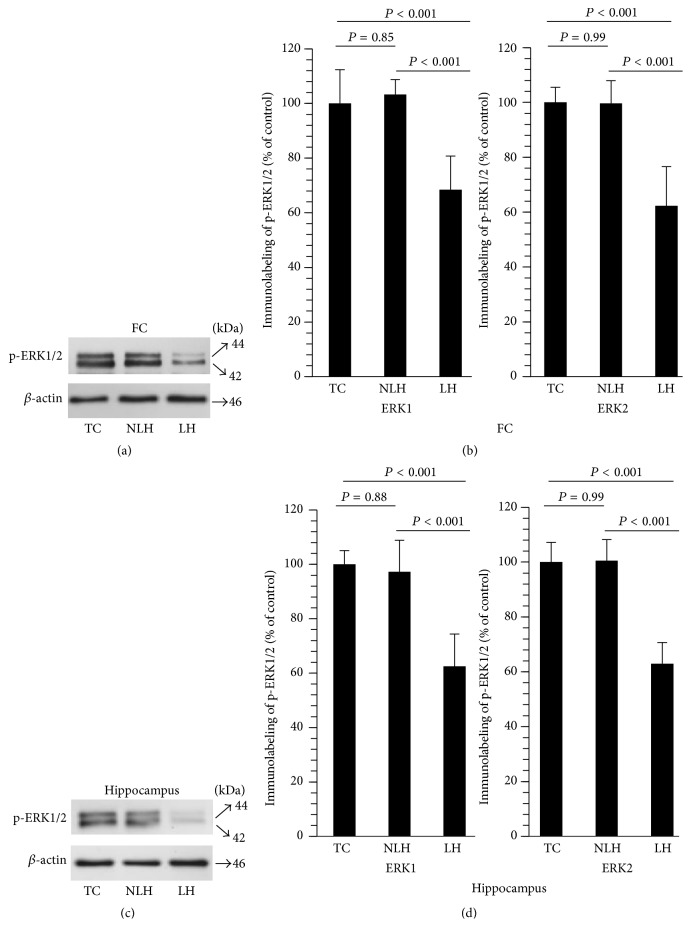
Immunolabeling of p-ERK1/2 in FC (a) and hippocampus (c) obtained from tested control (TC), non-learned helpless (non-LH), and learned helpless (LH) rats. *β*-actin was used as endogenous control. Mean ± SD of ERK1 and ERK2 phosphorylation in FC and hippocampus of TC, non-LH, and LH rats (*n* = 6/group) is depicted in (b) and (d), respectively. Overall group differences in the 3 groups are as follows. FC: ERK1, df = 2,15, *F* = 19.93, and *P* < 0.001; ERK2, df = 2,15, *F* = 28.04, and *P* < 0.001. Hippocampus: ERK1, df = 2,15, *F* = 26.12, and *P* < 0.001; ERK2, df = 2,15, *F* = 48.89, and *P* < 0.001.

To confirm whether the activity of ERK1 and ERK2 was associated with their altered phosphorylation forms, we measured the levels of p-ERK1/2 in the same tissue lysates of frontal cortex and hippocampus in which we determined ERK1 and ERK2 catalytic activities (Figures 6(a) and 6(c)). As with catalytic activity, phosphorylation of both ERK1 and ERK2 was significantly lower in LH rats (frontal cortex: Figure 6(b); hippocampus: Figure 6(d)). This phosphorylation was not altered in non-LH rats compared with TC rats in either brain area.

### 3.7. Correlation between Protein Expression Levels of ERK1 and ERK2 and Their Catalytic Activities

In the LH group, the catalytic activity of ERK1/2 was significantly correlated with protein expression of ERK1 in frontal cortex (cytosol: *r* = 0.71 and *P* = 0.001; nuclear: *r* = 0.85 and *P* < 0.001) and hippocampus (cytosol: *r* = 0.61 and *P* = 0.007; nuclear: *r* = 0.79 and *P* < 0.001). Similarly, catalytic activity of ERK1/2 was significantly correlated with protein levels of ERK2 in frontal cortex (cytosol: *r* = 0.82 and *P* < 0.001; nuclear: *r* = 0.70 and *P* = 0.001) and hippocampus (cytosol: *r* = 0.79 and *P* < 0.001; nuclear: *r* = 0.83 and *P* < 0.001) of LH rats.

### 3.8. ERK1/2-Mediated Phosphorylation of RSK1 and MSK1

**Figure 7:**
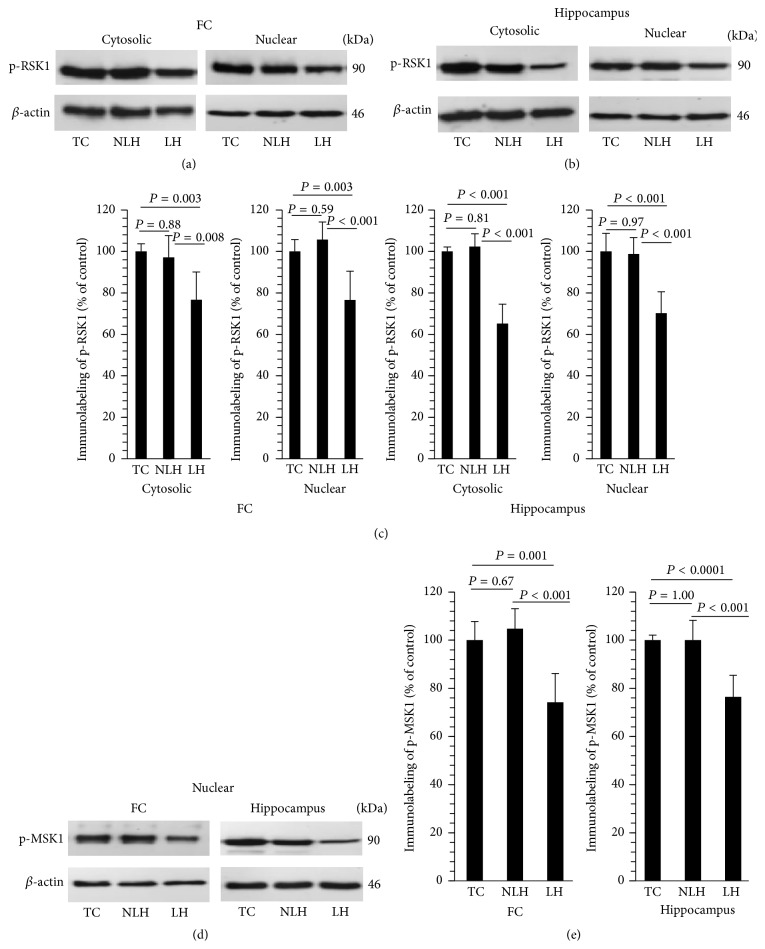
Representative Western blots showing ERK1/2-mediated phosphorylation of RSK1 in cytosolic and nuclear fractions of frontal cortex (FC) (a) and hippocampus (b) and MSK1 in nuclear fraction of FC and hippocampus (d) obtained from tested control (TC), non-learned helpless (non-LH), and learned helpless (LH) rats. *β*-actin was used as endogenous control. Differences in phosphorylation of RSK1 and MSK1 in FC and hippocampus between TC, non-LH, and LH rats are shown in (c) and (e), respectively. Data are the mean ± SD from 6 rats in each group. Overall group differences for RSK1 among TC, non-LH, and LH rats are as follows. FC: cytosolic, df = 2,15, *F* = 9.62, and *P* = 0.002; nuclear, df = 2,15, *F* = 14.42, and *P* < 0.001. Hippocampus: cytosolic, df = 2,15, *F* = 60.13, and *P* < 0.001; nuclear, df = 2,15, *F* = 20.86, and *P* < 0.001. Overall group differences for MSK1 in nuclear fraction among TC, non-LH, and LH rats are as follows. FC: df = 2,15, *F* = 17.73, and *P* < 0.001; hippocampus: df = 2,15, *F* = 21.70, and *P* < 0.001.

We examined ERK1/2-mediated phosphorylation of RSK1 in both cytosolic and nuclear fractions. ERK1/2-mediated phosphorylation of MSK1 was determined only in the nuclear fraction. Immunolabeling of p-RSK1 in the nuclear and cytosolic fractions of frontal cortex and hippocampus is shown in Figures 7(a) and 7(b), respectively. The levels of p-RSK1 were significantly lower in both cytosolic and nuclear fractions of frontal cortex and hippocampus obtained from LH rats (Figure 7(c)). Non-LH group did not show these changes in either frontal cortex or hippocampus compared with TC group. The Western blots of p-MSK1 in the nuclear fraction of frontal cortex and hippocampus are depicted in Figure 7(d) and are depicted as bar diagram in Figure 7(e). The level of MSK1 was significantly reduced in frontal cortex and hippocampus of LH rats without any change in non-LH rats.

### 3.9. Catalytic Activities of RSK1 and MSK1

**Figure 8:**
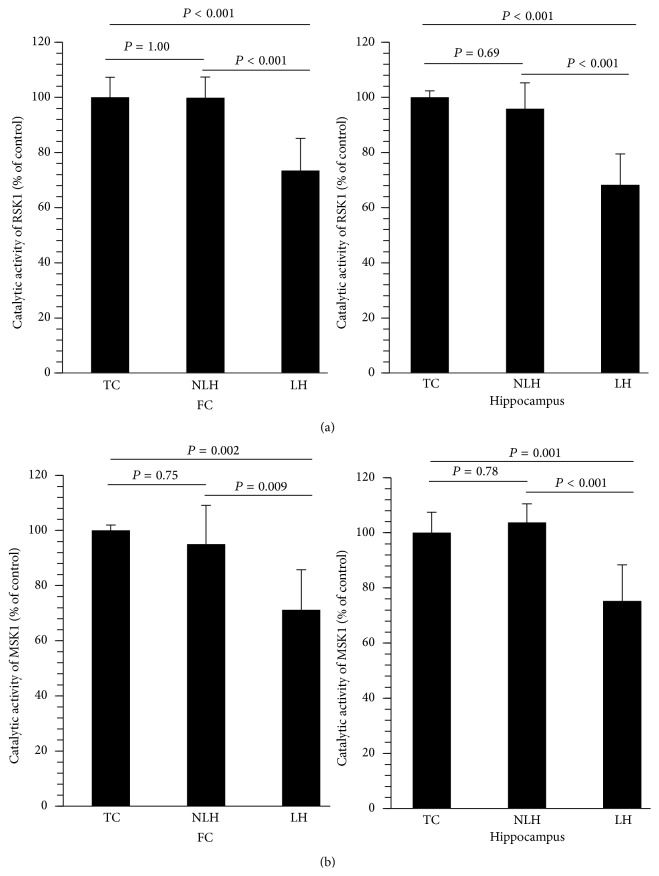
Catalytic activates of RSK1 and MSK1 in frontal cortex (FC) (a) and hippocampus (b) of tested control (TC), non-learned helpless (non-LH), and learned helpless (LH) rats. Overall group differences are as follows. RSK1: FC, df = 2,15, *F* = 17.21, and *P* < 0.001; hippocampus, df = 2,15, *F* = 24.38, and *P* < 0.001. MSK1: FC, df = 2,15, *F* = 10.21, and *P* < 0.002; hippocampus, df = 2,15, *F* = 15.75, and *P* = 0.12.

ERK1/2-mediated catalytic activities of RSK1 and MSK1 were determined in the tissue lysates of frontal cortex and hippocampus. Both RSK1 and MSK1 catalytic activities were significantly decreased in brain areas of LH rats (Figures 8(a) and 8(b)). These activities were unaltered in non-LH rats compared with TC rats.

### 3.10. mRNA and Protein Levels of RSK1 and MSK1

**Figure 9:**
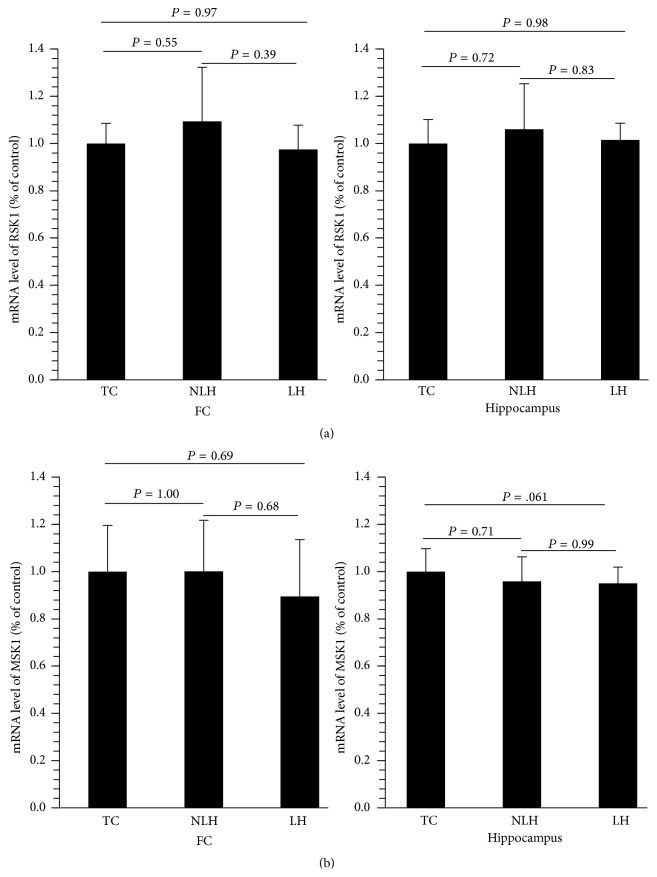
mRNA levels of RSK1 and MSK2 in frontal cortex (FC) (a) and hippocampus (b) of tested controls (TC), non-learned helpless (non-LH), and learned helpless (LH) rats. Data are the mean ± SD from 6 rats in each group. Overall group differences in the 3 groups are as follows. FC: df = 2,15, *F* = 1.00, and *P* = 0.39; hippocampus: df = 2,15, *F* = 0.33, and *P* = 0.72.

mRNA expressions of RSK1 or MSK1 were determined in the total fraction of frontal cortex and hippocampus. We did not find any significant change in the expression of RSK1 or MSK1 in the frontal cortex or hippocampus of any of the groups studied (Figures 9(a) and 9(b)).

**Figure 10:**
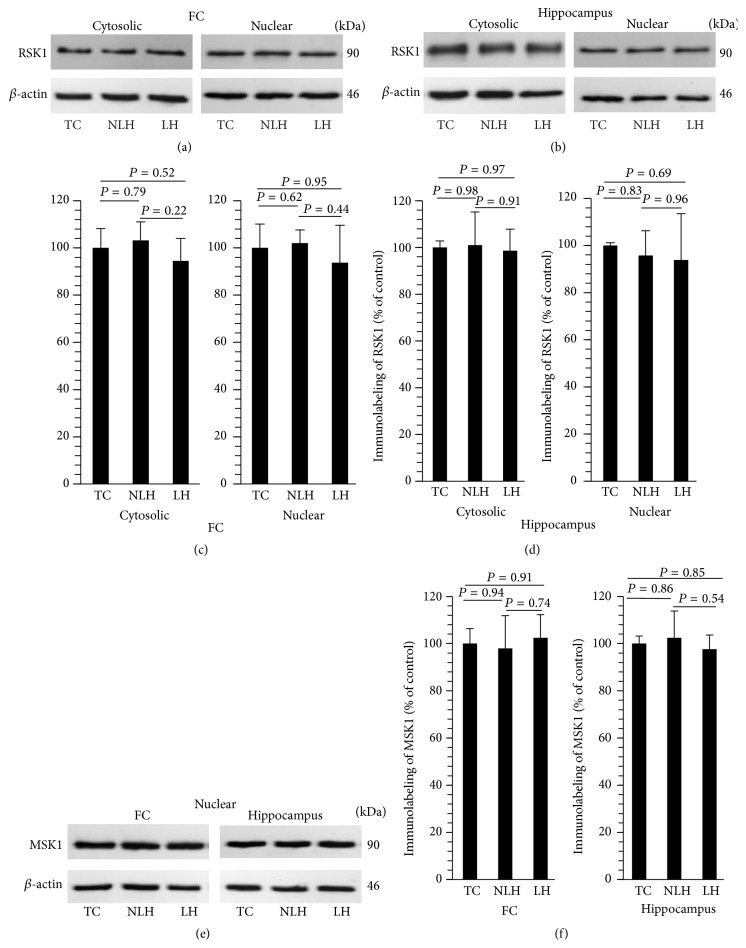
Representative immunoblots of total RSK1 in cytosolic and nuclear fractions of frontal cortex (FC) (a) and hippocampus (b) and MSK1 in nuclear fractions of FC and hippocampus (d) obtained from tested control (TC), non-learned helpless (non-LH), and learned helpless (LH) rats. *β*-actin was used as endogenous control. Differences in expression of RSK1 and MSK1 in FC and hippocampus between TC, non-LH, and LH rats are shown in (c) and (e), respectively. Data are the mean ± SD from 6 rats in each group. Overall group differences for RSK1 among TC, non-LH, and LH rats are as follows. FC, cytosolic: df = 2,15, *F* = 1.58, and *P* = 0.23; nuclear: df = 2,15, *F* = 0.85, and *P* = 0.44. Hippocampus, cytosolic: df = 2,15, *F* = 0.08, and *P* = 0.92; nuclear: df = 2,15, *F* = 0.36, and *P* = 0.70. Overall group differences for MSK1 in nuclear fraction among TC, non-LH, and LH rats are as follows. FC: df = 2,15, *F* = 0.27, and *P* = 0.76; hippocampus: df = 2,15, *F* = 0.58, and *P* = 0.57. No significant differences were found in expression of RSK1 or MSK1 in any brain area.

Protein levels of RSK1 were determined in cytosolic and nuclear fractions and those of MSK1 were determined in nuclear fraction of frontal cortex and hippocampus of TC, non-LH, and LH rats. Western blots of RSK1 in cytosolic and nuclear fractions of frontal cortex and hippocampus are shown in Figures 10(a) and 10(b), respectively. As with mRNA expression levels, no significant changes were found in the expression of RSK1 in cytosolic fraction or nuclear fraction of frontal cortex (Figure 10(c)) or hippocampus (Figure 10(d)) of LH rats. Similarly, the levels of MSK1 in nuclear fraction were unaltered in LH rats (Figures 10(e) and 10(f)).

### 3.11. Correlation between p-RSK1 and p-MSK1 with p-ERK1/2 Levels

The levels of p-RSK1 were significantly correlated with levels of p-ERK1/2 in frontal cortex (cytosolic: *r* = 0.54 and *P* = 0.02; nuclear: *r* = 0.69 and *P* = 0.002) and hippocampus (cytosolic: *r* = 0.76 and *P* < 0.001; nuclear: *r* = 0.87 and *P* < 0.002) of LH rats. Similarly, p-MSK1 and p-ERK1/2 were significantly correlated in the nuclear fraction of frontal cortex (*r* = 0.82; *P* < 0.001) and hippocampus (*r* = 0.77; *P* < 0.001) of LH rats.

## 4. Discussion

In the present study, we found that there is hypoactivation of ERK1/2 signaling in the brain of LH rats. This is based on several observations made in frontal cortex and hippocampus of LH rats compared with non-LH rats. For example, in LH rats, (1) catalytic activity of ERK1/2, measured as ERK1/2-mediated phosphorylation of Elk1, was significantly reduced; (2) phosphorylation and therefore the activation of ERK1 and ERK2 was significantly downregulated; (3) mRNA and protein expression of ERK1 and ERK2, measured independently, were significantly lower; (4) ERK1/2-mediated phosphorylation of downstream substrates RSK1 and MSK1 was significantly reduced; and (5) ERK1/2-mediated catalytic activities of both RSK1 and MSK1 were significantly lower. None of these changes were apparent in non-LH rats. It is pertinent to mention that our findings of reduced ERK1/2 signaling in the brain of LH rats represent abnormalities associated with prolonged depression as repeated stress paradigm used in the current study generated behavioral deficits that persisted for 14 days. In our earlier studies, we had found that single inescapable shock paradigm was sufficient to develop LH behavior; however, these behavioral deficits persisted only for 24 hours and were reversed thereafter [37, 38, 45]. In the future, it will be interesting to examine whether there is a differential regulation of ERK1/2 signaling in acute versus chronic depression as we have earlier reported in case of serotonergic receptors and associated signaling in LH rats [37, 38, 45], which may reflect adaptive versus maladaptive response.

Translocation of activated ERK1/2 is a crucial phenomenon in targeting substrates within the nucleus. Under resting conditions, ERK1 and ERK2 are primarily localized in cytosol [46]; however, once these kinases are phosphorylated by upstream MEK1 and MEK2, both ERK1 and ERK2 translocate to the nucleus [15]. Initially, we examined levels of activated (phosphorylated form) ERK1/2 in total tissue lysates and found that their activities were significantly reduced in LH rats without any change in non-LH rats. To further examine whether reduced phosphorylation of ERK1 and ERK2 was associated with alterations in their catalytic activities, we determined ERK1/2-mediated phosphorylation of Elk1 in cytosolic and nuclear fractions of frontal cortex and hippocampus. Interestingly, we found that the levels of p-Elk1 were lower in both these fractions, suggesting that the decrease in ERK1/2 activity is a generalized effect and may not be associated with translocation. This could be attributed to reduced expression of ERK1 and ERK2 as we have found in the brain of LH rats. ERK1 and ERK2 show a very close homology in amino acid sequences such that about 84% of amino acid residues are identical between these two kinases [43]. Also, the activation kinetics and substrate specificity for these two kinases are quite similar [47]. However, several studies point to subtle differences between these two kinases. For example, ERK1 knock-out mice are viable, but these mice show increased synaptic plasticity in the striatum brain area [48]. In contrast, ERK2 knock-out mice do not survive, suggesting that ERK2 deficiency is not compensated with ERK1 [49]. Also, at the cellular level, these two kinases regulate cell cycle in a different manner. Whereas ERK1 acts at G_2_/M level, ERK2 regulates G_1_ phase of the cell cycle [50]. In addition,* in situ* hybridization studies suggest that whereas ERK2 mRNA is expressed throughout the brain, ERK1 mRNA is confined to cortex, olfactory bulb, regions of hippocampus, amygdala, hypothalamus, and cerebellum. ERK1 mRNA is almost absent in the CA-1 area, whereas ERK2 is present in all neurons of the hippocampus [51, 52]. These studies suggest possible brain region-specific functions mediated by these two ERKs. In light of these observations, we examined the expression of both ERK1 and ERK2 in frontal cortex and hippocampus. We found that mRNA and protein levels of these two kinases were decreased in LH rats and that the degree of change was almost the same in these two brain areas. We also found significant correlations between ERK1 and ERK2 protein and mRNA levels with catalytic activity of ERK1/2. This indicates that lower activation of ERK1 and ERK2 could possibly be associated with less expression of these two isoforms of ERK.

A wide range of functions of ERK1 and ERK2 is mediated through phosphorylation of substrates. Among them, phosphorylation and activation of MAP kinase-activated kinases represent a crucial amplification step in the ERK1/2 cascade. Of these, RSKs and MSKs are directly regulated by ERK1/2. Four different isoforms of RSKs have been identified (RSKs 1–4) with close homology among themselves (~80%). As with ERK1 and ERK2, under resting conditions, RSKs reside in cytoplasm and translocate to the nucleus upon phosphorylation [53]. RSKs are highly expressed in the brain [54, 55] and participate in the regulation of cell cycle and in the proliferation and survival of neurons. The most important substrates of RSKs are serum response factor [56], CREB [57–59], and chromatin-associated histone H3 [60]. RSKs also interact with Ets transcription factors [61], which are required for activation of TIF-1A, a transcription initiation factor involved in the transcription of RNA polymerase I and synthesis of rRNAs. Activation of ERK1 and ERK2 promotes interaction between RSK and CBP, which along with p300 form nucleosome structure and participate in transcriptional activation [62]. RSKs also participate in the survival of neurons by phosphorylating and therefore deactivating a proapoptotic protein Bad [61]. In addition, death associated protein kinase, another apoptotic regulatory protein, is phosphorylated and deactivated upon activation of RSKs [63]. The other crucial substrates of ERK1/2 are MSKs. MSKs exist in two isoforms, MSK1 and MSK2, with ~75% amino acid homology [64]. Both MSK isoforms are highly expressed in the brain; however, at cellular level, unlike RSKs, MSKs are present in the nucleus. Upon activation, MSKs regulate gene transcription by phosphorylating transcription factors ATF-1 and CREB and increase the transcript stability by phosphorylating nuclear proteins [64–67]. In addition, MSKs phosphorylate proapoptotic Bad [68], Akt [69], and translational machinery component 4EBP1 [70]. To examine if reduced expression and activation of ERK1 and ERK2 lead to altered activation of RSKs and MSKs, we determined ERK1/2-mediated catalytic activities of RSK1 and MSK1 as well as ERK1/2-mediated phosphorylation of RSK1 and MSK1. We found that, in LH rats, catalytic activities of both RSK1 and MSK1 were reduced in frontal cortex and hippocampus. ERK1/2-mediated phosphorylation of RSK1 and MSK1 was also reduced in these brain areas of LH rats. None of these changes were apparent in non-LH rats. We examined the expression of RSK1 and MSK1 but did not find any significant change in their mRNA or protein levels. These results suggest that it is not the expression but the reduced activation of upstream ERK1 and ERK2 which contributes to the decreased phosphorylation and therefore activation of RSK1 and MSK1 in LH rats.

In our earlier postmortem brain studies, we have shown that expression and activation of ERK1 and ERK2 are reduced in various cortical and hippocampal brain areas of depressed patients but not in cerebellum [29]. We have also found that upstream kinases MEK1 and MEK2 were less active in these brain areas [30]. The present study confirms our human postmortem brain findings that indeed reduced ERK1/2 signaling is associated with depression. Similar to our human brain study, our preliminary study in LH, NLH, and TC rats demonstrates that ERK1/2 activation was affected in cerebellum (data not shown), suggesting brain region-specific changes in ERK1/2 in LH rats. Interestingly, several studies demonstrate that inhibition of ERK1/2 signaling causes impaired learning behavior in mice which is improved with increased ERK activity [71–73]. This appears to be relevant to the development of LH phenotype as these rats show learning deficit which could be associated with reduced ERK1/2 activation. On the other hand, ERK1/2 signaling remained unaltered in non-LH rats even though these rats were given the same stress paradigm as LH rats. This again shows that the development of resiliency towards depression could be dependent upon the status of ERK activation. In the future, it will be interesting to test whether overexpression of ERK1 or ERK2 in frontal cortex or hippocampus of LH rats can lead to non-LH behavior and whether reduced ERK1/2 activation in these brain areas of non-LH rats can induce LH phenotype. An earlier study has shown that systemic injection of MEK inhibitor resulted in reduced ERK phosphorylation and subsequent depressive-like behavior in rats [35]. This inhibition also blocked the effects of antidepressants in various behavioral tests [35]. This suggests that not only can ERK1/2 inhibition cause depression but reduced ERK1/2 can also block the effectiveness of antidepressants. This is further supported by observations that electroconvulsive shock induces activation and tyrosine phosphorylation of ERK1/2 in the rat hippocampus [74] and that fluoxetine reverses depressive-like behavior in rats by increasing ERK/CREB signaling [75].

The reason behind reduced expression and activation of ERK1 and ERK2 in LH rats is presently unclear; however, the possibility of the role of upstream regulators in altering ERK1/2 signaling cannot be ruled out. As is well known, ERK1 and ERK2 are activated by several G protein coupled receptors, receptors for tyrosine and nontyrosine kinases, and various effector molecules such as protein kinase A and protein kinase C either directly or via upstream kinases such as MAPK kinase kinases or Raf [11–13, 76]. Interestingly, neurotransmitter receptors such as 5HT_1A_ and 5HT_2A_ and *α*_2_ and *β*_2_ adrenergic receptors have been shown to be altered in the brain of depressed patients as well as in LH rats [38, 77, 78]. In addition, we have reported less activation of PKC and PKA not only in the brain of depressed patients [79, 80] but also in the frontal cortex and hippocampus of LH rats [37].

The consequence of reduced ERK1/2 signaling at functional levels in the brain of LH rats remains to be explored; however, as mentioned above, by phosphorylating several substrates either directly or indirectly, ERK1/2 can regulate transcription factors, stimulus-induced expression of immediately early genes, histone modifications, and translational machinery [81–83], which can lead to altered synaptic plasticity and physiological responses. In addition, since ERK1/2 is the major signaling pathway for BDNF-mediated response [84, 85] in promoting neuronal survival, proliferation, and differentiation [57], any abnormality in this signaling may also cause altered structural plasticity. It is pertinent to mention that neuronal atrophy of the prefrontal cortex and hippocampus has been reported in depressed patients [86–89], which could be associated with reduced BDNF levels and its mediated ERK1/2 activation [90]. Interestingly, it has been shown that lower ERK activity and reduced gray matter volume in depressed patients are related to depression associated risk allele Ser704Cyst [91] and that haplotypes and gene-gene interaction in the Ras/Raf/MAPK/RSK signaling pathway are involved in antidepressant remission in depressed population [92]. ERK1/2 pathway has been shown to exert its effects, in part, by regulating the synthesis of miRNA via increasing the stability of proteins belonging to the Argonaute complex, including dicer and the human immunodeficiency virus transactivation response RNA-binding protein (TRBP), which participate in the silencing of gene expression [93]. We recently reported that there is an adaptive miRNA response to inescapable shocks in non-LH rats, which was blunted in LH rats [39]. It will be interesting to examine whether differential activation of ERK1/2 has any impact on miRNAs response in LH or non-LH rats and in the development of these phenotypes.

In conclusion, we found differential responsiveness of ERK1/2 signaling in the brain of LH and non-LH rats. Whereas LH rats showed diminished activation and expression of ERK1 and ERK2 in frontal cortex and hippocampus, there was a muted response in non-LH rats. This was also evident at functional level where ERK1/2-mediated activation of RSK and MSK was lower in LH rats, without any change in non-LH rats. Our present and previous human postmortem brain studies [29–31] not only suggest that alterations in ERK1/2 may be important in the pathophysiology of depression but also raise the interesting possibility that ERK1/2 may be involved in generating vulnerability to depression phenotype. Follow-up studies will be needed to further investigate whether manipulation of ERK1/2 in these brain areas can induce or reverse LH phenotype.

## References

[1] Murray C. J. L., Lopez A. D. (1996). Evidence-based health policy—lessons from the global burden of disease study. *Science*.

[2] Kessler R. C., Berglund P., Demler O. (2003). The epidemiology of major depressive disorder: results from the National Comorbidity Survey Replication (NCS-R). *The Journal of the American Medical Association*.

[3] Demyttenaere K., Bruffaerts R., Posada-Villa J. (2004). Prevalence, severity, and unmet need for treatment of mental disorders in the World Health Organization World Mental Health Surveys. *The Journal of the American Medical Association*.

[4] Wong M.-L., Licinio J. (2004). From monoamines to genomic targets: a paradigm shift for drug discovery in depression. *Nature Reviews Drug Discovery*.

[5] Nierenberg A. A., Fava M., Trivedi M. H. (2006). A comparison of lithium and T_3_ augmentation following two failed medication treatments for depression: a STAR∗D report. *The American Journal of Psychiatry*.

[6] Duman R. S., Malberg J., Nakagawa S., D'Sa C. (2000). Neuronal plasticity and survival in mood disorders. *Biological Psychiatry*.

[7] Duman R. S. (2002). Pathophysiology of depression: the concept of synaptic plasticity. *European Psychiatry*.

[8] Fossati P., Radtchenko A., Boyer P. (2004). Neuroplasticity: from MRI to depressive symptoms. *European Neuropsychopharmacology*.

[9] Manji H. K., Quiroz J. A., Sporn J. (2003). Enhancing neuronal plasticity and cellular resilience to develop novel, improved therapeutics for difficult-to-treat depression. *Biological Psychiatry*.

[10] Giovannini M. G. (2006). The role of the extracellular signal-regulated kinase pathway in memory encoding. *Reviews in the Neurosciences*.

[11] Fukunaga K., Miyamoto E. (1998). Role of MAP kinase in neurons. *Molecular Neurobiology*.

[12] Numakawa T., Suzuki S., Kumamaru E., Adachi N., Richards M., Kunugi H. (2010). BDNF function and intracellular signaling in neurons. *Histology and Histopathology*.

[13] Raman M., Chen W., Cobb M. H. (2007). Differential regulation and properties of MAPKs. *Oncogene*.

[14] Lin L.-L., Wartmann M., Lin A. Y., Knopf J. L., Seth A., Davis R. J. (1993). cPLA_2_ is phosphorylated and activated by MAP kinase. *Cell*.

[15] Chen R.-H., Sarnecki C., Blenis J. (1992). Nuclear localization and regulation of erk-and rsk-encoded protein kinases. *Molecular and Cellular Biology*.

[16] Marais R., Wynne J., Treisman R. (1993). The SRF accessory protein Elk-1 contains a growth factor-regulated transcriptional activation domain. *Cell*.

[17] Reichardt L. F. (2006). Neurotrophin-regulated signalling pathways. *Philosophical Transactions of the Royal Society B: Biological Sciences*.

[18] Ohira K., Hayashi M. (2009). A new aspect of the TrkB signaling pathway in neural plasticity. *Current Neuropharmacology*.

[19] Skaper S. D. (2008). The biology of neurotrophins, signalling pathways, and functional peptide mimetics of neurotrophins and their receptors. *CNS & Neurological Disorders—Drug Targets*.

[20] Day J. J., Sweatt J. D. (2011). Epigenetic mechanisms in cognition. *Neuron*.

[21] Ciccarelli A., Giustetto M. (2014). Role of ERK signaling in activity-dependent modifications of histone proteins. *Neuropharmacology*.

[22] Kolkova K., Novitskaya V., Pedersen N., Berezin V., Bock E. (2000). Neural cell adhesion molecule-stimulated neurite outgrowth depends on activation of protein kinase C and the Ras-mitogen-activated protein kinase pathway. *Journal of Neuroscience*.

[23] Yang S.-H., Sharrocks A. D., Whitmarsh A. J. (2013). MAP kinase signalling cascades and transcriptional regulation. *Gene*.

[24] Weeber E. J., Sweatt J. D. (2002). Molecular neurobiology of human cognition. *Neuron*.

[25] Samuels I. S., Saitta S. C., Landreth G. E. (2009). MAP'ing CNS development and cognition: an ERKsome process. *Neuron*.

[26] Stornetta R. L., Zhu J. J. (2011). Ras and Rap signaling in synaptic plasticity and mental disorders. *Neuroscientist*.

[27] Alessi D. R., Gomez N., Moorhead G., Lewis T., Keyse S. M., Cohen P. (1995). Inactivation of p42 MAP kinase by protein phosphatase 2A and a protein tyrosine phosphatase, but not CL100, in various cell lines. *Current Biology*.

[28] Gopalbhai K., Meloche S. (1998). Repression of mitogen-activated protein kinases ERK1/ERK2 activity by a protein tyrosine phosphatase in rat fibroblasts transformed by upstream oncoproteins. *Journal of Cellular Physiology*.

[29] Dwivedi Y., Rizavi H. S., Roberts R. C., Conley R. C., Tamminga C. A., Pandey G. N. (2001). Reduced activation and expression of ERK1/2 MAP kinase in the post-mortem brain of depressed suicide subjects. *Journal of Neurochemistry*.

[30] Dwivedi Y., Rizavi H. S., Conley R. R., Pandey G. N. (2006). ERK MAP kinase signaling in post-mortem brain of suicide subjects: differential regulation of upstream Raf kinases Raf-1 and B-Raf. *Molecular Psychiatry*.

[31] Dwivedi Y., Rizavi H. S., Zhang H., Roberts R. C., Conley R. R., Pandey G. N. (2009). Aberrant extracellular signal-regulated kinase (ERK)1/2 signalling in suicide brain: role of ERK kinase 1 (MEK1). *International Journal of Neuropsychopharmacology*.

[32] Malki K., Pain O., Tosto M. G., Du Rietz E., Carboni L., Schalkwyk L. C. (2015). Identification of genes and gene pathways associated with major depressive disorder by integrative brain analysis of rat and human prefrontal cortex transcriptomes. *Translational Psychiatry*.

[33] Einat H., Yuan P., Gould T. D. (2003). The role of the extracellular signal-regulated kinase signaling pathway in mood modulation. *Journal of Neuroscience*.

[34] Engel S. R., Creson T. K., Hao Y. (2009). The extracellular signal-regulated kinase pathway contributes to the control of behavioral excitement. *Molecular Psychiatry*.

[35] Duman C. H., Schlesinger L., Kodama M., Russell D. S., Duman R. S. (2007). A role for MAP kinase signaling in behavioral models of depression and antidepressant treatment. *Biological Psychiatry*.

[36] Seligman M. E. P., Beagley G. (1975). Learned helplessness in the rat. *Journal of Comparative and Physiological Psychology*.

[37] Dwivedi Y., Mondal A. C., Shukla P. K., Rizavi H. S., Lyons J. (2004). Altered protein kinase A in brain of learned helpless rats: effects of acute and repeated stress. *Biological Psychiatry*.

[38] Dwivedi Y., Mondal A. C., Payappagoudar G. V., Rizavi H. S. (2005). Differential regulation of serotonin (5HT)2A receptor mRNA and protein levels after single and repeated stress in rat brain: role in learned helplessness behavior. *Neuropharmacology*.

[39] Smalheiser N. R., Lugli G., Rizavi H. S. (2011). MicroRNA expression in rat brain exposed to repeated inescapable shock: differential alterations in learned helplessness vs. non-learned helplessness. *International Journal of Neuropsychopharmacology*.

[40] Lowry O. H., Rosebrough N. J., Farr A. L., Randall R. J. (1951). Protein measurement with Folin phenol reagent. *The Journal of Biological Chemistry*.

[41] Dwivedi Y., Mondal A. C., Rizavi H. S., Conley R. R. (2005). Suicide brain is associated with decreased expression of neurotrophins. *Biological Psychiatry*.

[42] Sapkota G. P., Kieloch A., Lizcano J. M. (2001). Phosphorylation of the protein kinase mutated in Peutz-Jeghers cancer syndrome, LKB1/STK11, at Ser^431^ by p90^RSK^ and cAMP-dependent protein kinase, but not its farnesylation at Cys^433^, is essential for LKB1 to suppress cell growth. *The Journal of Biological Chemistry*.

[43] Boulton T. G., Cobb M. H. (1991). Identification of multiple extracellular signal-regulated kinases (ERKs) with antipeptide antibodies. *Cell Regulation*.

[44] Ortiz J., Harris H. W., Guitart X., Terwilliger R. Z., Haycock J. W., Nestler E. J. (1995). Extracellular signal-regulated protein kinases (ERKs) and ERK kinase (MEK) in brain: regional distribution and regulation by chronic morphine. *The Journal of Neuroscience*.

[45] Dwivedi Y., Mondal A. C., Rizavi H. S., Shukla P. K., Pandey G. N. (2005). Single and repeated stress-induced modulation of phospholipase C catalytic activity and expression: role in LH behavior. *Neuropsychopharmacology*.

[46] Reszka A. A., Seger R., Diltz C. D., Krebs E. G., Fischer E. H. (1995). Association of mitogen-activated protein kinase with the microtubule cytoskeleton. *Proceedings of the National Academy of Sciences of the United States of America*.

[47] Shaul Y. D., Seger R. (2007). The MEK/ERK cascade: from signaling specificity to diverse functions. *Biochimica et Biophysica Acta—Molecular Cell Research*.

[48] Mazzucchelli C., Vantaggiato C., Ciamei A. (2002). Knockout of ERK1 MAP kinase enhances synaptic plasticity in the striatum and facilitates striatal-mediated learning and memory. *Neuron*.

[49] Saba-El-Leil M. K., Vella F. D. J., Vernay B. (2003). An essential function of the mitogen-activated protein kinase Erk2 in mouse trophoblast development. *EMBO Reports*.

[50] Liu X., Yan S., Zhou T., Terada Y., Erikson R. L. (2004). The MAP kinase pathway is required for entry into mitosis and cell survival. *Oncogene*.

[51] Thomas K. L., Hunt S. P. (1993). The regional distribution of extracellularly regulated kinase-1 and -2 messenger RNA in the adult rat central nervous system. *Neuroscience*.

[52] Fiore R. S., Bayer V. E., Pelech S. L., Posada J., Cooper J. A., Baraban J. M. (1993). p42 mitogen-activated protein kinase in brain: prominent localization in neuronal cell bodies and dendrites. *Neuroscience*.

[53] Zhao Y., Bjørbæk C., Weremowicz S., Morton C. C., Moller D. E. (1995). RSK3 encodes a novel pp90rsk isoform with a unique N-terminal sequence: growth factor-stimulated kinase function and nuclear translocation. *Molecular and Cellular Biology*.

[54] Yntema H. G., van den Helm B., Kissing J. (1999). A novel ribosomal S6-kinase (RSK4; RPS6KA6) is commonly deleted in patients with complex X-linked mental retardation. *Genomics*.

[55] Zeniou M., Ding T., Trivier E., Hanauer A. (2002). Expression analysis of RSK gene family members: the RSK2 gene, mutated in Coffin-Lowry syndrome, is prominently expressed in brain structures essential for cognitive function and learning. *Human Molecular Genetics*.

[56] Brüning J. C., Gillette J. A., Zhao Y. (2000). Ribosomal subunit kinase-2 is required for growth factor-stimulated transcription of the c-Fos gene. *Proceedings of the National Academy of Sciences of the United States of America*.

[57] Bonni A., Brunet A., West A. E., Datta S. R., Takasu M. A., Greenberg M. E. (1999). Cell survival promoted by the Ras-MAPK signaling pathway by transcription-dependent and -independent mechanisms. *Science*.

[58] Ginty D. D., Bonni A., Greenberg M. E. (1994). Nerve growth factor activates a Ras-dependent protein kinase that stimulates c-fos transcription via phosphorylation of CREB. *Cell*.

[59] Xing J., Ginty D. D., Greenberg M. E. (1996). Coupling of the RAS-MAPK pathway to gene activation by RSK2, a growth factor-regulated CREB kinase. *Science*.

[60] Strelkov I. S., Davie J. R. (2002). Ser-10 phosphorylation of histone H3 and immediate early gene expression in oncogene-transformed mouse fibroblasts. *Cancer Research*.

[61] Wu J., Janknecht R. (2002). Regulation of the ETS transcription factor ER81 by the 90-kDa ribosomal S6 kinase 1 and protein kinase A. *The Journal of Biological Chemistry*.

[62] Nakajima T., Fukamizu A., Takahashi J. (1996). The signal-dependent coactivator CBP is a nuclear target for pp90_RSK_. *Cell*.

[63] Anjum R., Roux P. P., Ballif B. A., Gygi S. P., Blenis J. (2005). The tumor suppressor DAP kinase is a target of RSK-mediated survival signaling. *Current Biology*.

[64] Deak M., Clifton A. D., Lucocq J. M., Alessi D. R. (1998). Mitogen- and stress-activated protein kinase-1 (MSK1) is directly activated by MAPK and SAPK2/p38, and may mediate activation of CREB. *The EMBO Journal*.

[65] Arthur J. S. C., Cohen P. (2000). MSK1 is required for CREB phosphorylation in response to mitogens in mouse embryonic stem cells. *FEBS Letters*.

[66] Wiggin G. R., Soloaga A., Foster J. M., Murray-Tait V., Cohen P., Arthur J. S. C. (2002). MSK1 and MSK2 are required for the mitogen- and stress-induced phosphorylation of CREB and ATF1 in fibroblasts. *Molecular and Cellular Biology*.

[67] Schuck S., Soloaga A., Schratt G., Arthur J. S. C., Nordheim A. (2003). The kinase MSK1 is required for induction of c-fos by lysophosphatidic acid in mouse embryonic stem cells. *BMC Molecular Biology*.

[68] She Q.-B., Wei-Ya M., Zhong S., Dong Z. (2002). Activation of JNK1, RSK2, and MSK1 is involved in serine 112 phosphorylation of bad by ultraviolet B radiation. *The Journal of Biological Chemistry*.

[69] Nomura M., Kaji A., Ma W.-Y. (2001). Mitogen- and stress-activated protein kinase 1 mediates activation of Akt by ultraviolet B irradiation. *The Journal of Biological Chemistry*.

[70] Liu G., Zhang Y., Bode A. M., Ma W.-Y., Dong Z. (2002). Phosphorylation of 4E-BP1 is mediated by the p38/MSK1 pathway in response to UVB irradiation. *The Journal of Biological Chemistry*.

[71] Atkins C. M., Selcher J. C., Petraitis J. J., Trzaskos J. M., Sweatt J. D. (1998). The MAPK cascade is required for mammalian associative learning. *Nature Neuroscience*.

[72] Schafe G. E., Atkins C. M., Swank M. W., Bauer E. P., Sweatt J. D., LeDoux J. E. (2000). Activation of ERK/MAP kinase in the amygdala is required for memory consolidation of pavlovian fear conditioning. *Journal of Neuroscience*.

[73] Adams J. P., Sweatt J. D. (2002). Molecular psychology: roles for the ERK MAP kinase cascade in memory. *Annual Review of Pharmacology and Toxicology*.

[74] Berman D. E., Hazvi S., Rosenblum K., Seger R., Dudai Y. (1998). Specific and differential activation of mitogen-activated protein kinase cascades by unfamiliar taste in the insular cortex of the behaving rat. *Journal of Neuroscience*.

[75] Qi X., Lin W., Li J. (2008). Fluoxetine increases the activity of the ERK-CREB signal system and alleviates the depressive-like behavior in rats exposed to chronic forced swim stress. *Neurobiology of Disease*.

[76] Roberson E. D., English J. D., Adams J. P., Selcher J. C., Kondratick C., Sweatt J. D. (1999). The mitogen-activated protein kinase cascade couples PKA and PKC to cAMP response element binding protein phosphorylation in area CA1 of hippocampus. *Journal of Neuroscience*.

[77] Mann J. J. (2013). The serotonergic system in mood disorders and suicidal behaviour. *Philosophical Transactions of the Royal Society of London Series B: Biological sciences*.

[78] Flügge G., Van Kampen M., Mijnster M. J. (2004). Perturbations in brain monoamine systems during stress. *Cell and Tissue Research*.

[79] Pandey G. N., Dwivedi Y., Pandey S. C., Conley R. R., Roberts R. C., Tamminga C. A. (1997). Protein kinase C in the postmortem brain of teenage suicide victims. *Neuroscience Letters*.

[80] Pandey G. N., Dwivedi Y., Pandey S. C. (1999). Low phosphoinositide-specific phospholipase C activity and expression of phospholipase C *β*
_1_ protein in the prefrontal cortex of teenage suicide subjects. *The American Journal of Psychiatry*.

[81] Chandramohan Y., Droste S. K., Arthur J. S. C., Reul J. M. H. M. (2008). The forced swimming-induced behavioural immobility response involves histone H3 phospho-acetylation and c-Fos induction in dentate gyrus granule neurons via activation of the N-methyl-D-aspartate/extracellular signal-regulated kinase/mitogen- and stress-activated kinase signalling pathway. *European Journal of Neuroscience*.

[82] Laorden M. L., Núñez C., Almela P., Milanés M. V. (2002). Morphine withdrawal-induced c-fos expression in the hypothalamic paraventricular nucleus is dependent on the activation of catecholaminergic neurones. *Journal of Neurochemistry*.

[83] Valjent E., Pagès C., Hervé D., Girault J.-A., Caboche J. (2004). Addictive and non-addictive drugs induce distinct and specific patterns of ERK activation in mouse brain. *European Journal of Neuroscience*.

[84] Cavanaugh J. E., Ham J., Hetman M., Poser S., Yan C., Xia Z. (2001). Differential regulation of mitogen-activated protein kinases ERK1/2 and ERK5 by neurotrophins, neuronal activity, and cAMP in neurons. *The Journal of Neuroscience*.

[85] Segal R. A., Greenberg M. E. (1996). Intracellular signaling pathways activated by neurotrophic factors. *Annual Review of Neuroscience*.

[86] Sheline Y. I., Wang P. W., Gado M. H., Csernansky J. G., Vannier M. W. (1996). Hippocampal atrophy in recurrent major depression. *Proceedings of the National Academy of Sciences of the United States of America*.

[87] Öngür D., Drevets W. C., Price J. L. (1998). Glial reduction in the subgenual prefrontal cortex in mood disorders. *Proceedings of the National Academy of Sciences of the United States of America*.

[88] Rajkowska G. (2000). Postmortem studies in mood disorders indicate altered numbers of neurons and glial cells. *Biological Psychiatry*.

[89] Rajkowska G., Miguel-Hidalgo J. J., Wei J. (1999). Morphometric evidence for neuronal and glial prefrontal cell pathology in major depression. *Biological Psychiatry*.

[90] Dwivedi Y. (2009). Brain-derived neurotrophic factor: role in depression and suicide. *Neuropsychiatric Disease and Treatment*.

[91] Hashimoto R., Numakawa T., Ohnishi T. (2006). Impact of the DISC1 Ser704Cys polymorphism on risk for major depression, brain morphology and ERK signaling. *Human Molecular Genetics*.

[92] Wang C.-J., Zhang Z.-J., Xu Z. (2013). Kinase gene haplotypes and gene-gene interactions in the Ras-Raf-MAPK signaling pathway: association with antidepressant remission. *International Clinical Psychopharmacology*.

[93] Paroo Z., Ye X., Chen S., Liu Q. (2009). Phosphorylation of the human microRNA-generating complex mediates MAPK/Erk signaling. *Cell*.

